# Chloroplast Genomes and Comparative Analyses among Thirteen Taxa within Myrsinaceae s.str. Clade (Myrsinoideae, Primulaceae)

**DOI:** 10.3390/ijms20184534

**Published:** 2019-09-13

**Authors:** Xiaokai Yan, Tongjian Liu, Xun Yuan, Yuan Xu, Haifei Yan, Gang Hao

**Affiliations:** 1 College of Life Sciences, South China Agricultural University, Guangzhou 510642, China; yanxk@stu.scau.edu.cn (X.Y.); xyuan187@163.com (X.Y.); 2 Key Laboratory of Plant Resources Conservation and Sustainable Utilization, South China Botanical Garden, Chinese Academy of Sciences, Guangzhou 510650, China; liutongjian@scbg.ac.cn (T.L.); xuyuan@scbg.ac.cn (Y.X.); 3 Center of Plant Ecology, Core Botanical Gardens, Chinese Academy of Sciences, Guangzhou 510650, China

**Keywords:** Myrsinoideae, plastome, hypervariable regions, phylogeny, substitution rate

## Abstract

The Myrsinaceae s.str. clade is a tropical woody representative in Myrsinoideae of Primulaceae and has ca. 1300 species. The generic limits and alignments of this clade are unclear due to the limited number of genetic markers and/or taxon samplings in previous studies. Here, the chloroplast (cp) genomes of 13 taxa within the Myrsinaceae s.str. clade are sequenced and characterized. These cp genomes are typical quadripartite circle molecules and are highly conserved in size and gene content. Three pseudogenes are identified, of which *ycf15* is totally absent from five taxa. Noncoding and large single copy region (LSC) exhibit higher levels of nucleotide diversity (*Pi*) than other regions. A total of ten hotspot fragments and 796 chloroplast simple sequence repeats (SSR) loci are found across all cp genomes. The results of phylogenetic analysis support the notion that the monophyletic Myrsinaceae s.str. clade has two subclades. Non-synonymous substitution rates (*d*_N_) are higher in housekeeping (HK) genes than photosynthetic (PS) genes, but both groups have a nearly identical synonymous substitution rate (*d*_S_). The results indicate that the PS genes are under stronger functional constraints compared with the HK genes. Overall, the study provides hypervariable molecular markers for phylogenetic reconstruction and contributes to a better understanding of plastid gene evolution in Myrsinaceae s.str. clade.

## 1. Introduction

According to the present circumscription, Primulaceae s.l. comprises Primuloideae, Myrsinoideae, Theophrastoideae, and Maesoideae [1,2]. In Myrsinoideae, there are ca. 1500 species in 49 genera [3], and previous molecular phylogenetic studies classified these species into five clades: Myrsinaceae s.str. (includes *Aegiceras* Gaertn. but not *Maesa* Forssk.), tribe Lysimachieae, *Cyclamen* L., *Ardisiandra* Hook. f., and *Coris* L. [3,4]. Most temperate genera in the Myrsinoideae subfamily are perennial herbs, whereas the woody genera are mainly tropical [3]. The Myrsinaceae s.str. clade is a tropical woody representative in Myrsinoideae—which contains only a few herbaceous taxa, such as several species in *Labisia* Lindl. and *Ardisia* subg. *Bladhia* (Thunb.) Mez—and shows high species diversity (ca. 1300 species).

In contrast to the well-studied herbaceous clade (such as Lysimachieae [5,6,7,8] or *Cyclamen* [9]), the generic limits and alignments in the woody clade (i.e., Myrsinaceae s.str. clade) are rather unclear [3,10]. The phylogenetic uncertainties within the woody clade are most likely due to the limited number of genetic markers and/or insufficient taxon sampling. To date, only eleven genera within the woody clade (which contains 39 genera in total [3]) have been sparsely sampled, and very few plastid molecular markers (such as *rbcL*, *trnL-F*, *accD*, *rpoB*, *matK*, and *psbA-trnH*) and nuclear ribosomal DNA (Internal transcribed spacer region of nuclear ribosomal DNA, ITS) were employed by previous studies [9,10,11]. Thus, it is necessary to strengthen the genomic basis of this clade for phylogenetic analyses and biodiversity inventory.

Chloroplast (cp) markers such as *rbcL*, *matK*, and *trnL-trnF* have been widely used in phylogenetic studies [12,13] as well as the phylogeography [14,15] and the identification of plant species [16,17] owing to their high copy numbers within a cell, uniparental inheritance feature, and moderate evolutionary rate. However, the limited number of chloroplast markers has prevented us from resolving the phylogenetic relationships among closely related taxa and plant groups that have undergone rapid radiations [18,19].

Compared with traditional cp markers, complete cp genome sequences can improve phylogenetic resolution and have been successfully used to infer phylogenetic relationships among taxa [12,20,21,22]. Cp genome is also an ideal candidate for high-throughput sequencing and assembly due to its small size, conserved sequence and structure, and high cellular copy number [23]. Therefore, the number of complete cp genomes has rocketed in the recent years with the advancement of high-throughput DNA sequencing technologies [24].

Until now, only two cp genomes (*Ardisia polysticta* Miq. and *Ardisia crenata* Sims) of the Myrsinaceae s.str. clade were available within GenBank. The evolutionary patterns of the cp genomes within this clade are yet to be uncovered. Therefore, this study aims to (1) sequence, assemble, and characterize the cp genomes of the 13 woody taxa within clade Myrsinaceae s.str., and (2) probe into the evolutionary patterns of the cp genomes within this clade by estimating substitution rates of protein-coding genes.

## 2. Results and Discussion

### 2.1. Chloroplast Genomes Features

**Figure 1:**
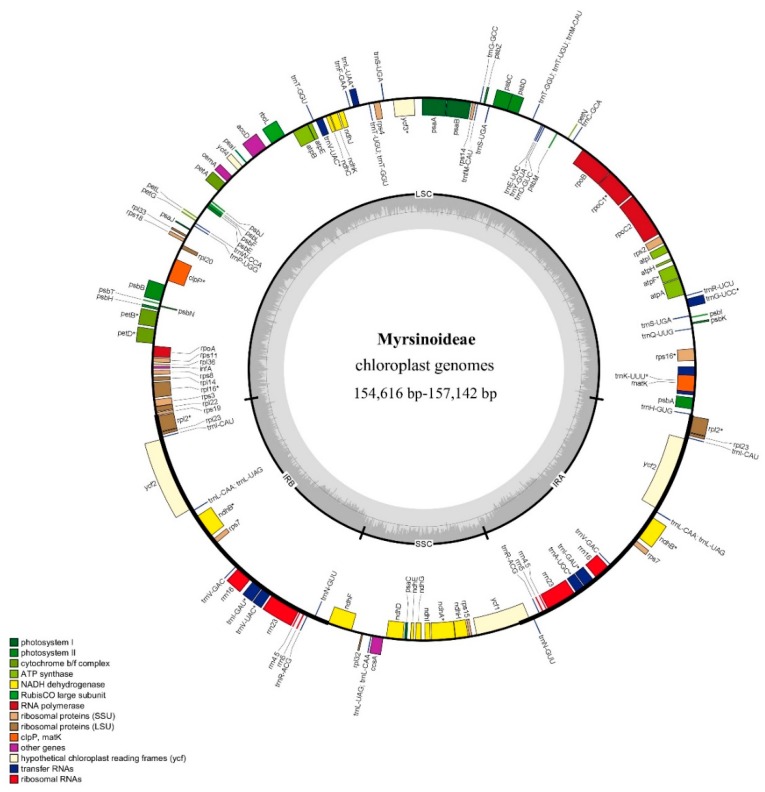
Map of the chloroplast genomes of Myrsinoideae species. Genes on the outer circle are transcribed in the counterclockwise direction, and those on the inner circle are transcribed in the clockwise direction. Bars of different colors indicate different functional groups.

**Figure 2:**
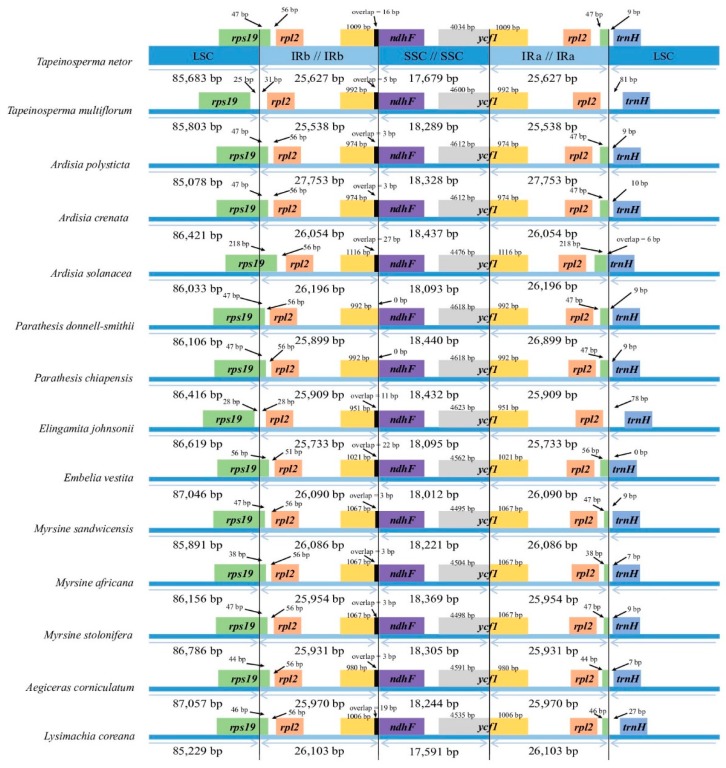
Comparisons of the boundaries between large single-copy (LSC), small single-copy (SSC), and inverted repeat (IR) regions among different Myrsinoideae cp genomes.

The cp genome sizes of the 13 taxa range from 154,616 bp (*Tapeinosperma netor* Guillaumin) to 157,241 bp [*Aegiceras corniculatum* (L.) Blanco) (Figure 1; Table 1]. All cp genomes are typical quadripartite circle molecules consisting of a pair of inverted repeat regions (IRs) ranging from 26,196 bp [*Ardisia solanacea* (Poir.) Roxb.] to 25,538 bp [*Tapeinosperma multiflorum* (Gillespie) A.C. Sm.] separated by a large single copy region (LSC) ranging from 87,057 bp (*Ae. corniculatum*) to 85,683 bp (*T. netor*) and a small single copy region (SSC) varying from 18,440 bp (*Parathesis donnell-smithii* Mez) to 17,679 bp (*T. netor*). Boundaries of the LSC, the IR, and the SSC regions are shown in Figure 2. The *rps19* and the *ycf1* genes both reside at the boundary of the IR and the SC regions. Their truncated copies are found in each of the IR regions (Figure 2). Total GC content ranges from 36.9% [in *Ae. coriniculatum*, *Embelia vestita* Roxb., and *Myrsine stolonifera* (Koidz.) E. Walker] to 37.1% (in *Ar. solanacea* and *T. netor*) (Table 1). The GC content of the IR regions—varying from 42.9% (in *Ae. coriniculatum*, *Em. vestita*, *Myrsine sandwicensis* A. DC.) to 43.2% (*Ar. solanacea*)—is higher than that of the LSC (about 34.8%) and the SSC (about 30.2%) regions (Table S1).

A total of 113–114 genes are identified from all thirteen cp genomes, among which 79–80 are protein-coding genes, 30 are tRNA genes, and four are rRNA genes. Five to six of the protein-coding genes, seven of the tRNA genes, and all four rRNA genes in the IR regions are duplicate genes. Sixteen genes (i.e., *atpF*, *ndhA*, *ndhB*, *petB*, *petD*, *rpl16*, *rpl2*, *rpoC1*, *rps12*, *rps16*, *trnA^UGC^*, *trnC^ACA^*, *trnE^UUC^*, *trnK^UUU^*, *trnL^UAA^*, and *trnS^CGA^*) contain a single intron, and two genes (*clpP* and *ycf3*) have two introns. Cp genome features of the woody taxa of Primulaceae are comparable to those of their herbal counterparts, namely, *Lysimachia* [25] and *Primula* [26].

Gene loss or pseudogenization of protein-coding genes in plastomes are common in angiosperms [27,28]. In the present study, three genes (*infA*, *accD*, and *ycf15*) are inferred to be pseudogenes in some taxa within clade Myrsinaceae s.str. (Table S1), which reoccurs in many plant lineages [28]. In Primulaceae, *accD* and *infA* pseudogenization has been reported in *Primula* species [26]. Moreover, *accD* is completely absent from three *Primula* taxa—*Primula kwangtungensis* W.W.Sm., *Primula persimilis* G. Hao, C.M. Hu & Y. Xu, and *Primula sinensis* Sabine ex Lindl. [26,29]. In seed plants, the *accD* and the *infA* genes transfer from the chloroplast to the nucleus genome [27], which is likely associated with their pseudogenization under relaxed purifying selection in plastome.

**Figure 3:**
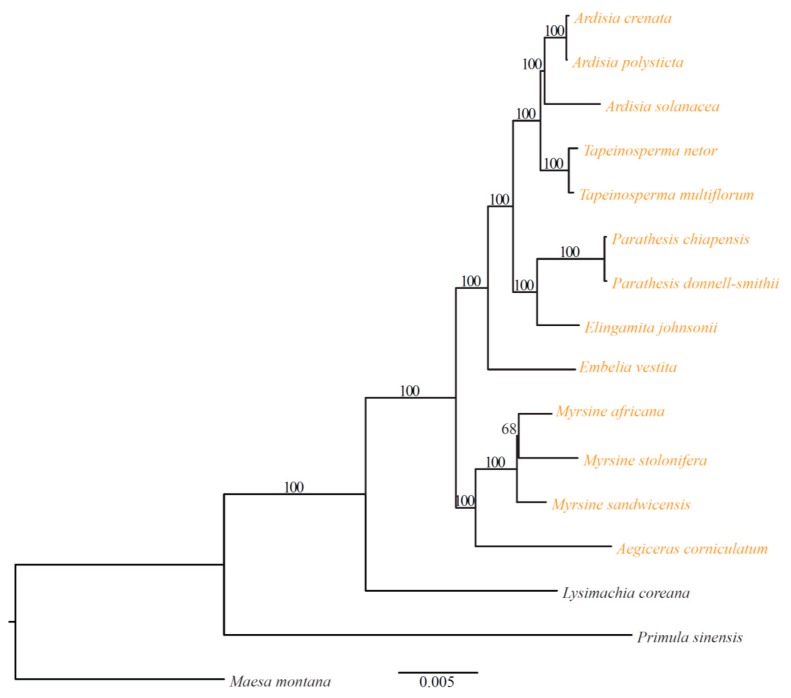
Maximum likelihood (ML) phylogenetic tree of Myrsinoideae based on 78 protein-coding genes. Numbers on the branches are bootstrap values. Species in the Myrsinaceae s.str. clade is indicated by yellow.

The *ycf15* gene, which displays a small open reading frame (ORF) in tobacco, has been pseudogenized in several angiosperm lineages (e.g., [30,31,32,33]). Some studies assumed that the *ycf15* may have originated from a non-functional intergenic sequence [30,33]. We find that *ycf15* is absent from five taxa, namely, *Elingamita johnsonii* G.T.S.Baylis, *T. multiflorum*, *T. netor*, *Pa. donnell-smithii* and *Parathesis chiapensis* Fernald, and has pseudogenized in the remaining taxa (Table S1). Based on the phylogenetic distribution of *ycf15*, we speculate that it may have lost several times in clade Myrsinaceae s.str. (Figure 3). To our knowledge, this is the first report of *ycf15* gene loss in Primulaceae [25,26], though its underlying mechanisms remain unknown.

### 2.2. Hypervariable Regions and CpSSRs

The sequence identities of 13 cp genomes of the Myrsinaceae s.str. clade are plotted using mVISTA (Figure S1), which shows that these genomes are relatively conserved. Noncoding (intergenic spacers and introns) sequences and the LSC regions often exhibit higher nucleotide substitution rates than other regions in plastome [27]. In this study, a total of ten highly variable regions with *Pi* > 0.03 (i.e., *trnK^UUU^-rps16*, *rps16-trnQ^UUG^*, *trnS^CGA^* intron, *petN-psbM*, *accD*, *rpl22-rps19*, *ndhF-rpl32*, *rpl32-trnL^UAG^*, *ccsA-ndhD*, and *ycf1*) are identified, among which the two most variable regions are *petN-psbM* (0.0457) and *trnK^UUU^-rps16* (0.0414). As shown by Jansen and Ruhlman [27], all ten mutation hotspots are located in the SC regions, six of which are in the LSC region. In addition, the introns with *Pi* > 0.03 all reside in the LSC region.

**Figure 4:**
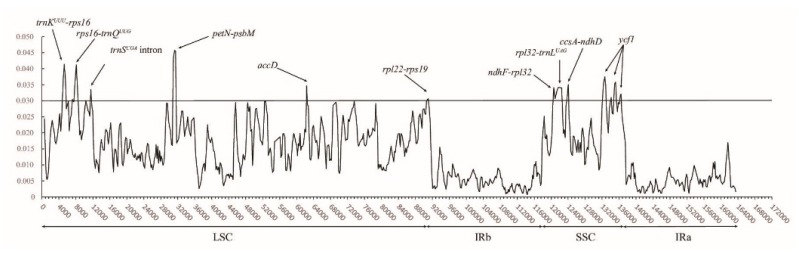
Nucleotide diversity (*Pi*) values across all Myrsinaceae s.str. cp genomes detected by sliding windows. *X*-axis, nucleotide positions in the cp genomes; *Y*-axis, nucleotide diversity (*Pi*) values. LSC: large single copy region; SSC: small single copy region; IR: inverted repeat region.

The *Pi* values of the protein-coding genes vary from 0.0007 to 0.0375. Only two genes, *accD* and *ycf1*, exhibit high *Pi* values (>0.03). As in many other angiosperms lineages, *ycf1* (5043–5610 bp in length) has two main divergence hotspots in clade Myrsinaceae s.str. (Figure 4) [34]. As a pseudogene in clade Myrsinaceae s.str., the relatively high nucleotide diversity of *accD* is presumably a result of relaxed selective pressure or the effects of Muller’s ratchet [35,36].

When compared with the results of Shaw et al. [37], the three hypervariable regions (*rps16-trnQ^UUG^*, *ndhF-rpl32*, and *rpl32-trnL^UAG^*) identified here are among the highest-ranking variable regions in all 25 lineages of angiosperms. In addition, *rbcL*, *trnL-F*, *accD*, *rpoB*, *matK*, and *psb-trnH* were previously employed in the phylogenetic analyses of Myrsinoideae [9,10,11]. The highest *Pi* value is 0.0198 for *rbcL*, 0.0201 for *trnL-F*, 0.0346 for *accD*, 0.0163 for *rpoB*, 0.0239 for *matK*, and 0.0244 for *psbA-trnH*. Only *accD* is among the top ten variable regions for the Myrsinaceae s.str. clade in our study. Overall, these high variable cp regions detected here can be employed for constructing the phylogeny and the phylogeographic inferences of Myrsinoideae by future studies.

**Figure 5:**
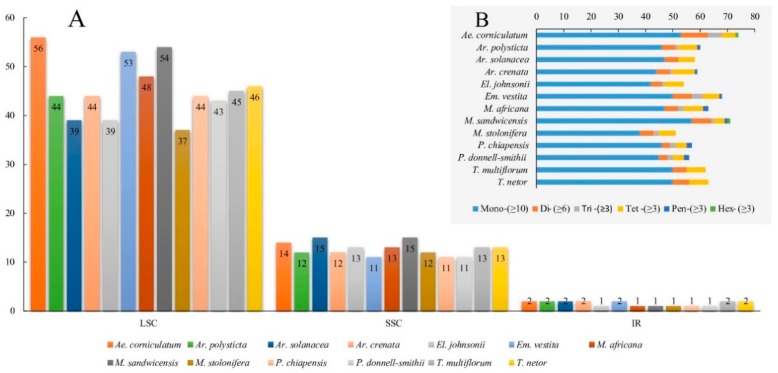
The type and the distribution of chloroplast simple sequence repeats (cpSSRs) across all Myrsinaceae s.str. cp genomes. (**A**) The number of cpSSRs identified in different species. (**B**) cpSSR distribution in different species.

Simple sequence repeats (SSR) are useful markers for population genetic studies [38]. Chloroplast SSR markers (cpSSRs) have emerged as excellent tools in population genetics owing to their unique non-recombination and uniparental inheritance characteristics [39]. A total of 796 SSR loci with six types (i.e., mono-, di-, tri-, tetra-, penta-, and hexa-nucleotides) are identified in the 13 Myrsinaceae s.str. cp genomes. These cpSSRs are mainly located in the LSC region (74.37%), followed by the SSC (20.73%) and the IR (2.51%) regions (Figure 5). Among all cpSSRs identified in this study, most are mono-nucleotide repeats (77.26%), followed by tetr-nucleotide repeats (9.80%) and di-nucleotide repeats (8.80%) (Figure 5). Other types, namely, tri-nucleotide, penta-nucleotide, and hexa-nucleotide repeats, account for 2.64%, 1.26%, and 0.25% of all cpSSRs, respectively. In addition, seven cpSSR loci are shared across all 13 species in the Myrsinaceae s.str. clade (Table S2). 

### 2.3. Phylogenetic Relationships

Clade Myrsinaceae s.str. contains ca. 39 genera and 1300 species and is the largest group of Primulaceae [3]. However, the generic limits and alignments of the Myrsinaceae s.str. clade are still unclear. In this study, phylogenetic analysis was performed based on 78 protein-coding genes (PCGs) in Myrsinoideae cp genomes using *Lysimachia coreana* Nakai, *Pr. sinensis*, and *Maesa montana* A. DC. as outgroups (Figure 3). Our phylogenetic results support the notion that clade Myrsinaceae s.str. is monophyletic and consists of two subclades. All *Myrsine* taxa and *Ae. corniculatum* cluster into the first subclade, and the remaining genera including *Ardisia*, *Tapeinosperma*, *Parathesis*, *Elingamita* and *Embelia* fall into the other clade (Figure 3). Almost all branches of the phylogenetic tree are strongly supported, as indicated by Figure 3. In addition, our results reveal that *Aegiceras* is closer to *Myrsine* but not to *Ardisia* and its allies (see [9]).

### 2.4. Substitution Rates and Their Variations among Genes

Substitution rates often vary considerably among genes in the cp genome [40]. Previous studies have reported a higher synonymous substitution rate (*d*_S_) than non-synonymous substitution rate (*d*_N_) due to natural selection [41]. Similarly, we find that the average *d*_N_ values of the cp genes vary from 0.0013 to 0.1143 with a mean value of 0.0305, and the average *d*_S_ values range from 0.0156 to 0.3445 with a mean value of 0.1096. In addition, the *d*_N_ and the *d*_S_ of each gene group vary from 0.0052 to 0.0806 (mean value: 0.0295) and 0.0327 to 0.1561 (mean value: 0.1054), respectively. Consistent with that observed in angiosperms [42], the mean synonymous substitution rate of the gene group is approximately three times faster than that of the non-synonymous substitution rate.

**Figure 6:**
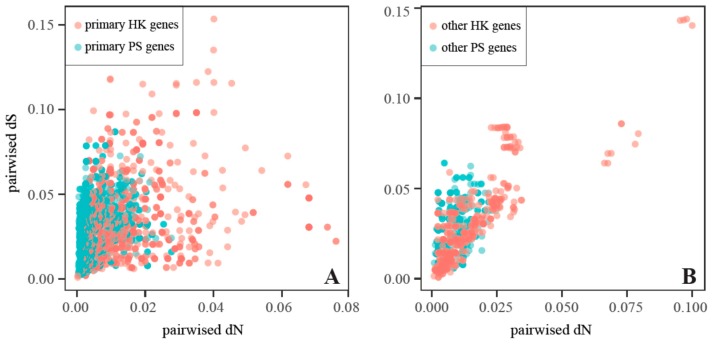
Bivariate plots of pairwise synonymous (*d*_S_) and nonsynonymous (*d*_N_) substitution rates of the protein-coding genes from the Myrsinaceae s.str. cp genomes. (**A**) Bivariate plot of the *d*_N_ vs. the *d*_S_ of primary housekeeping (HK) and photosynthetic (PS) genes, (**B**) bivariate plot of the *d*_N_ vs. the *d*_S_ of the other HK and PS genes.

Substitution rate variation between the housekeeping (HK) and the photosynthetic (PS) genes in the cp genome was reported by Wicke and Schneeweiss [41]. We observe a significant difference in pairwise substitution rate between the primary HK and the primary PS genes (or between the other HK and other PS genes) across all 13 Myrsinaceae s.str. taxa (*p* < 0.001 based on non-parametric Wilcoxon rank sum tests). Both bivariate plots indicate that the rate distribution of HK genes is broader than that of PS genes (Figure 6), suggesting relatively lower purifying selection pressure on the HK genes than on the PS genes.

**Figure 7:**
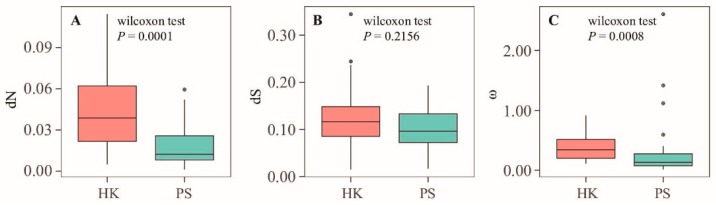
Comparison of the *d*_S_, the *d*_N_, and the ω values between the chloroplast primary PS and HK genes within clade Myrsinaceae s.str.; (**A**) boxplot of the *d*_N_ values, (**B**) boxplot of the *d*_S_ values, (**C**) boxplot of the ω values. The *p*-values were calculated by non-parametric Wilcoxon rank sum tests in R.

The boxplots in Figure 7 show that the *d*_N_ values of the primary HK genes are significantly higher than those of the primary PS genes (non-parametric Wilcoxon rank sum tests, *p* < 0.001), whereas the *d*_S_ values show no significant difference between the two categories (*p* = 0.2156; Figure 7). Thus, the PS genes have a lower *d*_N_/*d*_S_ ratio compared with the HK genes (non-parametric Wilcoxon rank sum tests, *p* < 0.001, Figure 7), re-enforcing the idea that the PS genes are under stronger functional constraints than the HK genes [43].

The *d*_N_/*d*_S_ ratio is an important indicator of selective pressure at the protein level, with ω > 1 suggesting positive selection [44]. However, positive selection is reasonable if *d*_S_ summed over all branches on the tree is >0.5 (PAML FAQ, http://saf.bio.caltech.edu/saf_manuals/pamlFAQs.pdf). Here, the individual *d*_N_/*d*_S_ ratios of *psbK*, *petG*, and *psaJ* are 2.6062, 1.1178, and 1.4159, respectively, but their *d*_S_ values summed over all branches on the tree are extremely low—0.0199, 0.0257, and 0.0295 for *psbK*, *petG*, and *psaJ*, respectively. Accordingly, the high *d*_N_/*d*_S_ ratios of the three genes are likely caused by insufficient mutation signals rather than positive selection.

## 3. Materials and Methods

### 3.1. Sampling

In the present study, plant (or DNA) samples of the thirteen woody species representing eight genera within clade Myrsinaceae s.str. were collected. The chloroplast genome sequences of two species (*Ar. polysticta* and *Ar. crenata*) were downloaded from GenBank (Table 1). Of the remaining eleven species, the DNAs of five species—*Ar. solanacea, El. johnsonii, My. africana, My. sandwicensis*, and *T. netor*—were generously provided by Kew DNA Bank of Royal Botanic Gardens, Kew (http://dnabank.science.kew.org/), and plant materials of the other six species were collected from the field and specimens. Refer to Table 1 for details on sample collection. Cp genomes of the thirteen woody species in clade Myrsinaceae s.str. were obtained and analyzed. *L. coreana* (clade Lysimachieae, Myrsinoideae; KM819521), *Pr. sinensis* (Primuloideae; KU321892), and *Ma. montana* (Maesoideae; KU569490), whose cp genomes are publicly available in GenBank, were used as outgroups for the phylogenetic analyses.

### 3.2. DNA Extraction and Sequencing

Total genomic DNA was extracted from dry leaves using a modified cetyltrimethyl ammonium bromide (CTAB) method [45]. DNA concentration was determined using the Qubit Fluorometer (Thermo Fisher Scientific, Waltham, MA, USA). The short-insert (ca. 500 bp) paired-end libraries were prepared using the TruePrepTM DNA Library Prep Kit V2 for Illumina (Vazyme Biotech Co., Ltd., Nanjing, China) following the manufacturer’s protocols. The libraries were sequenced on Illumina Hiseq X Ten platform (Illumina, Inc., San Diego, CA, USA) at Beijing Genomics Institute (Shenzhen, China) with read length of 150 bp. Raw sequencing data were qualified by FastQC v0.11.7 (http://www.bioinformatics.babraham.ac.uk/projects/fastqc/), and the adaptor sequences were removed by Trimmomatic 0.36 [46]. Approximately 2 Gb clean reads for each taxon were obtained.

### 3.3. Chloroplast Genome Assembly and Annotation

The clean sequences were assembled into complete cp genomes by NOVOPlasty 2.6.3 [47] using the cp genome of *Ar. polysticta* (Genbank No. NC_021121) as the reference with a default k-mer of 39. For samples that failed to yield complete cp genomes, we assembled the reads into scaffolds using Spades 3.11.1 [48]. The locations of scaffolds were determined using Blast 2.7.1 (http://blast.ncbi.nlm.nih.gov/) for generating a complete consensus sequence using the “Map to reference” function implemented in Geneious v11.0.3 [49].

Complete cp genomes were annotated using DOGMA v1.2 [50], and tRNAs were annotated using ARAGORN [51]. The raw annotations were subsequently examined and adjusted manually based on the reference cp genome (NC_021121) in Geneious (v11.0.3) to determine gene structures. The four SC-IR junctions in all cp genomes were examined for whether expansions or contractions of the IR regions had occurred. The complete annotated cp genome sequences were deposited in GenBank (Table 1). Circular cp genome maps were drawn using OrganellarGenome DRAW [52] with default settings and checked manually.

### 3.4. Identification of CpSSRs and Hypervariable Regions

Chloroplast simple sequence repeats (cpSSRs) were identified using MISA [53]. The minimum number of repeats was set as 10, 5, 4, 3, 3, and 3 for mono-, di-, tri-, tetra-, penta-, and hexa-nucleotide SSRs, respectively.

To detect hypervariable regions, the sequences of all 13 complete cp genomes were aligned using MAFFT v7.308 [54]. The sliding window analysis was conducted to evaluate nucleotide diversity (*Pi*) using DnaSP v6.11.01 [55] with a window size of 600 bp and a step size of 200 bp. The overall sequence identity among these cp genomes was plotted using mVISTA program under shuffle-LAGAN mode [56] with a reference of *Ar. polysticta* cp genome.

### 3.5. Phylogenetic Analyses

The 78 sequences of the protein-coding genes (PCGs) were extracted from all 13 cp genomes and aligned using the translation align function in MAFFT v7.308 with the L-INS-I method in Geneious. The genetic code was set as “Bacterial”, and the translation frame was set to 1. The aligned matrixes were concatenated into a super matrix for phylogenetic reconstruction. The best partition scheme was analyzed using PartitionFinder 2 with greedy search under the AICc criterion [57]. The maximum likelihood (ML) method was used for phylogenetic reconstruction by RAxML-HPC v8.2.20 [58] via CIPRES Science Gateway [59]. The ML analysis was performed with the GTR + Γ model under the best partitioning scheme. Node supports were evaluated by 1000 rapid bootstrap replicates. *L. coreana*, *Pr. sinensis*, and *Ma. montana* were used as outgroups.

### 3.6. Substitution Rate Estimation

To investigate the evolutionary patterns of the cp genomes, the rates of nonsynonymous (*d*_N_) and synonymous (*d*_S_) substitutions as well as the *d*_N_/*d*_S_ ratios were determined using PAML’s codeml [60] with codon frequencies F3 × 4. Gaps were excluded using cleandata = 1 to avoid spurious rate inference [61]. The *d*_N_/*d*_S_ ratios were estimated by excluding genes with *d*_S_ value smaller than 0.001. Genes without nonsynonymous and/or synonymous mutations were also excluded from further statistical analyses. The ML tree constructed based on the cp protein-coding genes was used as a constraint tree. The shared protein-coding genes within clade Myrsinaceae s.str. were classified into the following categories: (1) primary housekeeping (HK) genes (*rpo*, *rpl*, and *rps*), primary photosynthetic (PS) genes (*atp*, *ndh*, *pet*, *psa,* and *psb*), other HK genes (*clpP*, *matK*, *ycf1,* and *ycf2*), and other PS genes (*ccsA*, *cemA*, *rbcL*, *ycf3*, and *ycf4*) according to the criteria described by Wicke et al. [62]; (2) concatenated gene sets for the functional groups; and (3) all individual genes [61]. Statistical analyses were performed using R 3.6.1 [63].

## 4. Conclusions

Here, we sequenced and characterized the complete cp genomes of 13 taxa within the Myrsinaceae s.str. clade of Primulaceae. These cp genomes are highly conserved in terms of size and gene content and are typical quadripartite circle molecules consisting of an LSC region, an SSC region, and a pair of separated IRs. Three genes (*infA*, *accD*, and *ycf15*) were inferred to be pseudogenes, and *ycf15* was completely absent from five taxa. The noncoding regions (intergenic spacers and introns) and the LSC regions showed relatively higher nucleotide diversity (*Pi*) values than other regions. A total of ten hypervariable regions were identified. A total of 796 cpSSR loci under six types were found across the 13 cp genomes, 74.37% of which were found to be located in the LSC region. Results with the phylogenetic analyses suggest that Myrsinaceae s.str. is a monophyletic clade that contains two main subclades. The HK genes exhibited a significantly higher *d*_N_ compared with the PS genes, whereas the *d*_S_ values of HK and PS genes were similar. These results indicate that the PS genes underwent stronger functional constraints than the HK genes.

## Figures and Tables

**Table 1:** Details on samples, vouchers, GenBank accessions, and features of the chloroplast genomes within the Myrsinaceae s.str. clade.

Taxon	Voucher No./Herbarium Code	GenBank Accession	Size (bp)	GC Content	Gene No.	Protein Coding Gene	tRNA	rRNA
*Aegiceras comiculatum*	Liu150016/IBSC	MN167882	157,241	36.9%	114	80	30	4
*Ardisia solanacea*	17988*/K	MN094783	156,518	37.1%	114	80	30	4
*Ardisia polysticta*	No data	KC465962	156,506	37.1%	113	80	29	4
*Aridisa crenata*	Yxk160038/IBSC	KM719568	156,876	37.1%	113	80	30	4
*Elingamita johnsonii*	958*/K	MN094784	156,180	37.0%	113	79	30	4
*Embelia vestita*	Liu150050/IBSC	MN167884	157,238	36.9%	114	80	30	4
*Myrsine africana*	30087*/K	MN165129	156,433	37.0%	114	80	30	4
*Myrsine sandwicensis*	38322*/HAW	MN177700	156,284	37.0%	114	80	30	4
*Myrsine stolonifera*	Liu150044/IBSC	MN167883	156,953	36.9%	114	80	30	4
*Parathesis chiapensis*	Alush Nendes 6574/ARIZ	MN177699	156,666	37.0%	113	79	30	4
*Parathesis donnell-smithii*	Alvaro Campos 3924/ARIZ	MN177698	156,344	37.0%	113	79	30	4
*Tapeinosperma multiflorum*	9361/MO	MN177701	155,168	37.0%	113	79	30	4
*Tapeinosperma netor*	33984*/K	MN177702	154,616	37.1%	113	79	30	4

Note: * indicates the DNA identification (ID) number of the Royal Botanic Gardens Kew DNA Bank.

## Supplementary Materials

Supplementary materials can be found at https://www.mdpi.com/1422-0067/20/18/4534/s1.
