# Supplementary material for: Chloroplast Genomes and Comparative Analyses among Thirteen Taxa within Myrsinaceae s.str. Clade (Myrsinoideae, Primulaceae)
Source: Int J Mol Sci. 2019 Sep 13;20(18):4534. doi: 10.3390/ijms20184534 (PMC6769889; doi:10.3390/ijms20184534)
Supplement: Supplementary file 1 [file ijms-20-04534-s001.pdf]

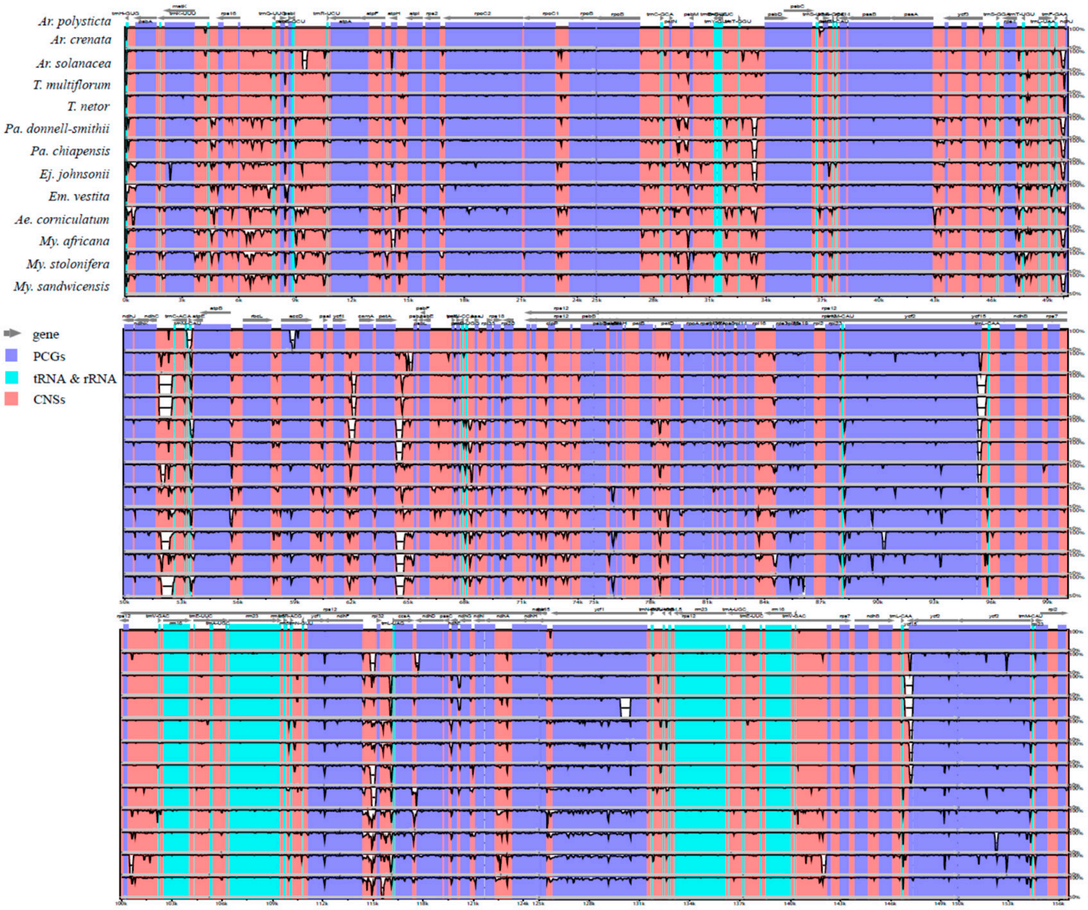

Figure S1. Visualization of alignment of the 13 cp genome sequences of Myrsinaceae s.str. clade. The cp genome of *Aridisa polysticta* is used as reference. Genome regions are color coded as protein-coding genes (PCGs), tRNAs and rRNAs, and conserved non-coding sequences (CNSs). The dark grey arrows indicate the annotated genes with their transcriptional direction. The percentage of identity is from 50% to 100% in vertical scale.

Table S1. Features of the cp genomes within the Myrsinaceae s.str. clade.

Taxon	LSC size (bp)	SSC size (bp)	IR size (bp)	GC content in LSC	GC content in SSC	GC content in IRs	*ψaccD* (bp)	*ψycf15* (bp)	*ψinfA* (bp)
Aegiceras comiculatum	87,057	18,244	25,970	34.7%	30.1%	42.9%	1,497	204	234
Aridisa crenata	86,421	18,347	26,054	34.9%	30.2%	43.0%	1,074	-	234
Ardisia polysticta	86,078	18,328	26,050	34.9%	30.2%	43.0%	1,494	204	234
Ardisia solanacea	86,033	18,093	26,196	34.9%	30.3%	43.2%	1,494	204	234
Elingamita johnsonii	86,619	18,095	25,733	34.8%	30.3%	43.1%	1,482	-	234
Embelia vestita	87,046	18,012	26,090	34.7%	30.2%	42.9%	1,494	204	234
Myrsine africana	86,156	18,369	25,954	34.8%	30.3%	43.0%	1,497	204	234
Myrsine sandwicensis	85,891	18,221	26,086	34.8%	30.1%	42.9%	1,497	204	234
Myrsine stolonifera	86,786	18,305	25,931	34.7%	30.3%	43.0%	1,497	204	234
Parathesis chiapensis	86,416	18,432	25,909	34.8%	30.1%	43.0%	1,494	-	234
Parathesis donnell-smithii	86,106	18,440	25,899	34.8%	30.1%	43.0%	1,494	-	234
Tapeinosperma multiflorum	85,803	18,289	25,538	34.9%	30.1%	43.1%	1,494	-	234
Tapeinosperma netor	85,683	17,679	25,627	34.9%	30.3%	43.1%	1,494	-	234

Table S2. Seven shared cpSSR loci across all cp genomes within the Myrsinaceae s.str. clade.

Repeat unit	Location
T	*trnK*^{UUU} intron
T	*rpo2*
T	*ndhC-trnC*^{ACA}
T	*rpoB*
C	*rps15*
AT	*rpo2*
AATA	*ndhD*
